# TNFAIP3 Plays a Role in Aging of the Hematopoietic System

**DOI:** 10.3389/fimmu.2020.536442

**Published:** 2020-11-03

**Authors:** Molly A. Smith, Ashley E. Culver-Cochran, Emmalee R. Adelman, Garrett W. Rhyasen, Averil Ma, Maria E. Figueroa, Daniel T. Starczynowski

**Affiliations:** ^1^ Experimental Hematology and Cancer Biology, Cincinnati Children’s Hospital Medical Center, Cincinnati, OH, United States; ^2^ Department of Cancer Biology, University of Cincinnati, Cincinnati, OH, United States; ^3^ Department of Human Genetics, University of Miami, Miami, FL, United States; ^4^ Department of Medicine, University of California, San Francisco, San Francisco, CA, United States; ^5^ Sylvester Comprehensive Cancer Center, University of Miami, Miami, FL, United States; ^6^ Department of Pediatrics, Cincinnati Children’s Hospital Medical Center, Cincinnati, OH, United States

**Keywords:** hematopoietic (stem) cells, aging, inflammation, A20 (TNFAIP3), hematopoiesis

## Abstract

Hematopoietic stem and progenitor cells (HSPC) experience a functional decline in response to chronic inflammation or aging. Haploinsufficiency of A20, or TNFAIP3, an innate immune regulator, is associated with a variety of autoimmune, inflammatory, and hematologic malignancies. Based on a prior analysis of epigenomic and transcriptomic changes during normal human aging, we find that the expression of A20 is significantly reduced in aged HSPC as compared to young HSPC. Here, we show that the partial reduction of A20 expression in young HSPC results in characteristic features of aging. Specifically, heterozygous deletion of A20 in hematopoietic cells resulted in expansion of the HSPC pool, reduced HSPC fitness, and myeloid-biased hematopoiesis. These findings suggest that altered expression of A20 in HSPC contributes to an aging-like phenotype, and that there may be a common underlying mechanism for diminished HSPC function between inflammatory states and aging.

## Introduction

Hematopoietic stem cells (HSCs) produce and maintain blood lineages that are responsible for tissue homeostasis and immunity. Two characteristics that make HSC essential to hematopoiesis are self-renewal and multilineage differentiation, the ability to differentiate into all blood cell types. In the steady state, HSC are quiescent and daily blood production is executed by hematopoietic progenitor cells. Upon injury or infection, inflammatory signals induce acute HSC proliferation to restore homeostasis (1, 2). Autoimmune disease, failure to resolve inflammation, or chronic inflammation results in adverse effects on HSC self-renewal and loss of potency, and is often accompanied by myeloid-biased differentiation (3–7). This functional decline of HSC is reminiscent of what occurs during aging (4, 8–12). In fact, there are many common features between aging hematopoiesis, chronic inflammation, and hematologic malignancies. Genome sequencing of human hematologic malignancies has revealed somatic mutations in inflammatory signaling genes, implicating inflammatory signaling in leukemogenesis.

A20, or *TNFAIP3*, an anti-inflammatory signaling molecule that restricts multiple intracellular signaling cascades, is important for hematopoiesis and HSC function (13, 14). A20 contains an OTU domain that functions as a deubiquitinating protease, and zinc finger domains that confer E3 ligase activity (15, 16). In response to several immune pathways, including Tumor Necrosis Factor Receptor, Interleukin-1 Receptor, and Toll-Like Receptor signaling, A20’s dual ubiquitin-editing functions negatively regulate excessive NF-κB signaling by modifying ubiquitin chains on key intermediates RIP1 and TRAF6 (16–18). Under normal cellular conditions, A20 is expressed at low levels, however, stimulation of pathogen associated molecular patterns (PAMPs) and pro-inflammatory cytokines acutely induce the expression of A20 in an NF-κB-dependent manner (19, 20). Genetic studies in humans have revealed a link between germline single nucleotide polymorphisms in *TNFAIP3* with the susceptibility to autoimmune and inflammatory diseases, such as systemic lupus erythematosus, rheumatoid arthritis, and Crohn’s disease (13, 21). Further, there is biallelic inactivation of A20 in one-third of patients with B-cell lymphomas, suggesting A20 may function as a tumor suppressor (22, 23). Consistent with these clinical observations, A20-deficient mice prematurely die of systemic inflammation, highlighting A20’s involvement in preventing inflammatory and autoimmune diseases (24).

During aging, HSCs increase in frequency, undergo myeloid-biased lineage priming and differentiation, and exhibit a functional decline in regeneration potential (9, 11, 12). Recent work has characterized the epigenomic and transcriptional changes during normal aging of human HSCs. Phenotypically defined HSCs (Lineage^-^CD34^+^CD38^-^) from aged individuals demonstrated epigenetic reprogramming including redistribution of DNA methylation and reduction in H3K27ac, H3K4me1, and H3K4me3 at regulatory elements of select genes as compared to HSC from young individuals (25). Although many genes, enhancers, and promoters are distinctly affected in young and aged HSCs, we found that A20 expression was significantly reduced in aged HSCs by ~40% as compared in young HSCs in humans and mice. Given that defects in A20 are linked to the pathogenesis of autoimmune disorders, aberrant hematopoiesis, and hematologic malignancies (13, 21), we posited that partially reduced expression of A20 may impact hematopoiesis and HSCs consistent with premature aging. While prior studies have generated conditionally targeted A20 deleted mice to study lineage-specific and tissue-specific deletions of A20, only a few studies have examined the loss of A20 in the hematopoietic system (26–28). Characterization of conditional A20 knockout models demonstrated myeloid proliferation, B cell apoptosis, and anemia accompanied by overproduction of pro-inflammatory cytokines (26). Bone marrow transplantation revealed that hematopoietic cells were the source of these abnormalities. Moreover, A20 has been directly implicated in HSC function. Deletion of A20 in hematopoietic cells using conditional and inducible mouse models revealed that complete loss of A20 in HSCs is incompatible with hematopoiesis (27, 28). These studies, which provide important insights into the role of A20 as a critical regulator of immune cell function and hematopoiesis, focused on the complete loss of A20 to illuminate the contribution of A20 on normal hematopoiesis. However, the consequences of partial reduction of A20 expression (i.e., as in heterozygous deletion of A20), which is seen aged HSCs and in autoimmune and inflammatory disorders, remains to be determined. In this study, conditional heterozygous deletion of A20 was examined in the hematopoietic system. We report that partial deletion of A20 results in expansion of an HSC-enriched pool, reduced hematopoietic stem and progenitor cell (HSPC) fitness, and myeloid-biased hematopoiesis. Though the phenotype of reduced A20 levels is more subtle than complete A20 loss, it is of clinical interest. Collectively, these findings suggest that altered expression of A20 in HSC contributes to an aging-like phenotype and represent a link between inflammatory disorders and aged hematopoiesis.

## Methods 

### Mice

All mice were bred, housed and handled in the Association for Assessment and Accreditation of Laboratory Animal Care-accredited animal facility of Cincinnati Children’s Hospital Medical Center (CCHMC). Animal care was in strict compliance with the institutional guidelines established by CCHMC, the Guide for the Care and Use of Animals (National Academy of Sciences 1996), and the Association for Assessment and Accreditation of Laboratory Animal Care International. A20^fl/fl^ mice were kindly provided by AM (University of California San Francisco) and described elsewhere (29). A20^fl/fl^ mice were crossed with *Vav*-Cre mice (Jackson Laboratory, 008610) or *Rosa*CreER mice (Jackson Laboratory, 008463) for conditional deletion of A20 (*A20* ^fl/fl^;*Vav*-Cre, *A20* ^fl/fl^;*Rosa*CreER). Both male and female mice were included in all experiments. BM cells were obtained by crushing the femur, tibia, and pelvic bone, and maintained in Iscove’s MDM (Cellgro, Cat No. 10-016-CV) with 10% fetal bovine serum.

### Tamoxifen Treatment

Conditional deletion of A20 was achieved in *A20* ^fl/fl^;*Rosa*CreER mice by intraperitoneal injection of 4 mg tamoxifen in corn oil. Bone marrow was harvested 5 days after tamoxifen treatment for *in vitro* analysis.

### Hematological and Histological Analysis

Peripheral blood was collected by retro-orbital bleeds or cardiac puncture. Blood counts were measured with an automated blood analyzer (HEMAVET). Blood smears and bone marrow cell cytospins were stained with Wright-Giemsa using a Hematek 3000 slide stainer (Siemens).

### BM Transplantation

For non-competitive BM transplantations, 3 x 10^6^ CD45.2^+^ BM mononuclear cells (MNC) from WT (C57Bl/6) and *A20^-/-^*VavCre were transplanted into lethally-irradiated recipient mice (CD45.1^+^ B6.SJL^Ptprca Pep3b/BoyJ^; 6–10 weeks of age). For competitive repopulation, 3 x 10^6^ CD45.2^+^ MNCs from WT (C57Bl/6) and *A20^-/-^*VavCre^+^ (C57Bl/6) mice (two donor mice, <3 months old) were mixed with 3 x 10^6^ CD45.1^+^ B6.SJL^PtprcaPep3b/BoyJ^ BM MNCs (6–10 weeks of age), and then transplanted into lethally-irradiated recipient mice (CD45.1^+^ B6.SJL^PtprcaPep3b/BoyJ^; 6–10 weeks of age), as previously described (30).

### Flow Cytometry

For immunophenotypic analysis of lineage positive cells, PB samples were processed with 1 x Pharm Lyse buffer (BD Biosciences), and then incubated with CD11b-PE-cy5 (15-0112-82, eBiosciences), Gr1-eFluor-450 (48-5931-82, eBiosciences), CD3-PE (12-0031-83, eBiosciences), and/or B220-APC (17-0452-82, eBiosciences). BM cells were stained with CD11b-PE-Cy5, Gr1-eFluor450, CD3-PE, B220-APC, Ter119-PE-Cy7 (25-5921-81, eBiosciences). To distinguish donor from recipient hematopoietic cells, PB were stained with CD45.1-PE-Cy7 (25-0453-82, eBiosciences) and CD45.2-APC-eFluor780 (47-0454-82, eBiosciences). For HSPC analysis, BM cells were washed and incubated for 30 min with biotin conjugated lineage markers (CD11b, Gr1, Ter119, CD3, B220, mouse hematopoietic lineage biotin panel, [88-7774-75 eBiosciences]), followed by staining with streptavidin eFluor450 (48-4317-82, eBiosciences), Sca-1-PE (12-5981-82, eBiosciences), c-Kit-APC-eFluor780 (47-1170-80, eBiosciences), CD48-PerCP-eFluor710 (46-0481-82, eBiosciences), CD150-APC (17-1501-81, eBiosciences), CD127-PE-Cy5 (15-1271081, eBiosciences), CD34-APC (17-0349-41,eBiosciences), CD16/32-PerCP-Cy5.5 (17-0161-81, eBioscience), CD45.1-PE-Cy7 (25-0453-82, eBiosciences), and CD45.2-FITC (561874, BD Biosciences). HSC were identified based on expression of Lin^-^Sca^-^1^+^c-Kit^+^CD150^+^CD48^-^ and CD150^-^LSK. Common myeloid progenitor cells (CMP) were identified base on expression of Lin^-^Sca^-^1^-^c-Kit^+^CD34^+^CD16/32^-^; megakaryocyte-erythroid progenitor cells (MEP) were identified base on expression of Lin^-^Sca^-^1^-^c-Kit^+^CD34^-^CD16/32^-^; granulocyte-macrophage progenitor cells (GMP) were identified based on expression of Lin^-^Sca^-^1^-^c-Kit^+^CD34^+^CD16/32^+^. To determine HSPC chimerism after competitive transplantation, BM cells were incubated with biotin conjugated lineage markers as described above, followed by staining with streptavidin eFluor450, Sca-1-PE, c-Kit-APC-eFluor780, CD48-PerCP-eFluor710, CD150-APC, CD45.1-PE-Cy7, and CD45.2-FITC. Analysis was performed using FACSCanto and/or LSRII flow cytometers and with either Diva or FlowJo software.

### Hematopoietic Progenitor Colony Assays

For colony forming assays in methylcellulose, 2.5 x 10^2^ murine HSPCs were plated in 1 ml of MethoCult^®^ GF M3434 (Stem Cell Technologies) per well in triplicate of an untreated 6-well culture plate. After 7 days, total colonies were counted and replated at 1 x 10^3^ cells per 1ml of MethoCult^®^ GF M3434 as previously described (31).

### RT-PCR and qRT-PCR

Total RNA was extracted and purified using Quick-RNA MiniPrep (Zymo research, R1055) and reverse transcription was carried out using SuperScript VILO cDNA Synthesis Kit (Invitrogen). Quantitative PCR was performed with Taqman Master Mix (Life Technologies) for *Tnfaip3* (Assay ID Mm00437121_m1) and *GAPDH* (Cat 4351370, Assay ID Hs02758991_g1).

### Cell Cycle Analysis

LSK BM cells were incubated with 10 μM EdU for 5 and 8 h, followed by fixation, permeabilization, and incubation with fluorescently conjugated antibody and 4’,6-diamidino-2-phenylindole according to the Click-iT Plus EdU Alexa Fluor 647 Flow Cytometry Assay kit protocol (Invitrogen, Cat No. C10634).

### Immunoblotting

LSK BM cells were separated into nuclear and cytoplasmic fractions. Briefly, to prepare the cytoplasmic lysate, cells were resuspended in 1 volume hypotonic solution with 0.1% Triton X-100, incubated on ice for 5 min, and centrifuged. The supernatant was collected and mixed with 1 volume of 5X SDS buffer. To prepare the nuclear lysate, the remaining pellet was washed twice with the hypotonic solution with 0.1% Triton X-100, resuspended in 2 volumes of 2.5X SDS buffer, and incubated on ice for 20 min. Lysates were incubated at 95°C for 5 min before separation by SDS-PAGE. Proteins were transferred to nitrocellulose membrane, blocked for 30 min with 5% non-fat dry milk in 0.5% TBS-T, and immunoblotted with antibodies raised against A20 (Cat No. 5630), p65 (Cat No. 8242), Lamin B1 (Cat No. 12586S), pIKKα/β (Cat No. 2697S), and actin (Cat No. 4968), all from Cell Signaling Technology. Immunoblots were probed with anti-rabbit HRP-conjugated antibody (eBiosciences, 18-8816-31), developed using ECL Western Blotting Substrate (Pierce, 32106), and imaged using a ChemiDoc Imaging System (Bio-Rad).

### Human Aging Data Set

As described by Adelman et al., raw RNA-seq counts from young (18–30 yo) and aged (65–75 yo) HSCe (Lineage^-^CD34^+^CD38^-^; n = 10 per age group) were generated using QoRTS (25, 32). Regularized log counts were then generated using DESeq2 v1.10.1 (33), with a multifactor design in order to control for sex of the donor as well as any batch effect during library preparation. ChIP-seq data from young and aged HSPC was visualized using the UCSC genome browser with tracks that were normalized by read count and to the IP’s corresponding Input (GEO accession GSE104404). Active TSS and Active Enhancers were defined as in Adelman et al., 2019.

### Statistical Analysis

Unless otherwise specified, results are depicted as the mean ± standard deviation. Statistical analyses were performed using Student’s t-test. For Kaplan-Meier analysis, Mantel-Cox test and hypergeometric test was used. GraphPad Prism (v5, GraphPad) was used for statistical analysis.

## Results

### A20 Expression Is Reduced in Aged HSC

**Figure 1:**
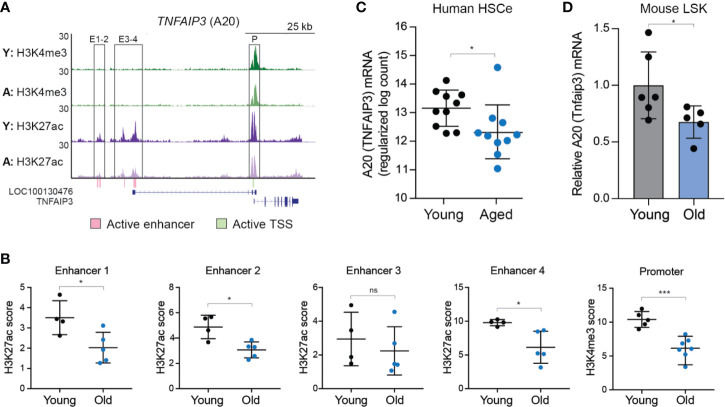
A20 expression is reduced in aged human and mouse HSC. **(A)** UCSC genome browser tracks of active enhancers within the TNFAIP3/A20 locus that overlap ChIP-seq peaks. Tracks are of pooled replicates for each age group, normalized to reads per million and to the corresponding input. Y, young; A, aged. **(B)** Quantification of the peak height from Figure 1A for H3K27ac within enhancers (E) 1-4 or H3K4me3 within the promoter (P). *P < 0.05; ***P < 0.001. **(C)** RNA-seq gene expression analysis of TNFAIP3 (A20) in young (n = 10) and aged (n = 10) HSCe (Lineage^-^CD34^+^CD38^-^). **(D)** *Tnfaip3 (A20)* mRNA expression in young and old LSK BM cells by qRT-PCR (n = 6 and 5 per group, respectively). *P < 0.05. Data are represented as mean ± standard deviation.

The level of A20 impacts the function of hematopoietic and immune cells. Therefore, we examined the expression levels of A20 in young and aged human HSC. As recently reported (25), an HSC-enriched lineage negative (Lin^-^) CD34^+^CD38^-^ (herein referred as HSCe) fraction was isolated from the bone marrow (BM) mononuclear cells collected from 10 young (18–30 years old) and 10 aged (65–75 years old) healthy donors and then evaluated for gene expression and epigenetic changes. In this data set, the enhancer regions (#1, 2, and 4) and transcriptional start site (TSS) neighboring A20/TNFAIP3 exhibited a significant reduction in H3K27Ac and H3K4me3, respectively, normally associated with transcriptional activation, in aged HSCe as compared to the young HSCe (**Figures 1A, B**). Furthermore, A20 expression was significantly reduced (P = 0.03) in the aged HSCe as compared to the young HSCe (**Figure 1C**). To determine whether reduced A20 expression is a conserved phenomenon in other species, we isolated HSC-enriched Lin^-^Sca1^+^cKit^+^ (LSK) from six young C57Bl/6 mice (10–11 weeks of age) and five old C57Bl/6 mice (between 33–87 weeks of age) and evaluated A20 mRNA expression by qRT-PCR. Consistent with aged HSCe from human donors, aged mouse HSPCs exhibit a reduction in A20 mRNA expression as compared to young mouse HSPCs (**Figure 1D**). Notably, the expression of A20 mRNA in the aged mouse HSPCs was approximately 40% lower than in the young HSPCs (**Figure 1D**). These findings revealed that A20 expression is reduced in phenotypically defined aged HSC and suggest that partial reduction in A20 expression may impact hematopoiesis.

### Complete Deletion of A20 Impairs Normal HSC Function and Hematopoiesis

To study the effect of A20 loss in the hematopoietic stem cell compartment, we first crossed *Tnfaip3*-floxed mice with VavCre^+^ transgenic mice, which have a transgene encoding Cre recombinase expressed specifically in all hematopoietic cells (herein referred to as A20^-/-^VavCre^+^) (**Supplemental Figure 1**). As previously reported, complete deletion of A20 in hematopoietic cells during embryonic development results in premature lethality, and for surviving mice, a marked reduction in body size (**Supplemental Figure 2A**). The surviving A20-deleted mice exhibited significant myeloid-biased differentiation, with mice primarily presenting with neutrophilia (P < 0.001) accompanied by a significant decrease in lymphocytes (P < 0.001) (**Supplemental Figure 2B**). Peripheral blood smears revealed ineffective erythropoiesis, as evidenced by poikilocytosis and spined cell morphology of red blood cells (RBC), as well as polychromasia (**Supplemental Figure 2C**). In accordance with peripheral blood observations, bone marrow pathology showed an expanded neutrophil population (**Supplemental Figure 2C**). Functional assessment of BM progenitor cells revealed that deletion of A20 resulted in reduced colony-forming potential in methylcellulose affecting all of the hematopoietic progenitor lineages (**Supplemental Figure 2D**). To determine the consequences of complete A20 deletion on adult hematopoiesis, BM cells isolated from WT (VavCre+) and surviving A20^-/-^VavCre^+^ were transplanted into lethally irradiated syngeneic recipient mice. All recipient mice engrafted with BM cells from A20^-/-^VavCre^+^ mice perished within 14 days post transplantation while all recipient control mice survived (**Supplemental Figure 2E**). Lastly, we performed competitive BM transplantation assays by mixing excess numbers of BM cells from A20^-/-^VavCre^+^ (CD45.2^+^) mice with CD45.1^+^ BoyJ mice (10:1 ratio). As expected, the hematopoietic chimerism of A20^-/-^VavCre^+^ BM cells was significantly diminished (P < 0.05), as evident by a marked reduction in engrafted CD45.2^+^ cells accompanied by an increase in CD45.1^+^ cells (**Supplemental Figure 2F**). These findings along with published observations indicate that complete loss of A20 is incompatible with normal hematopoiesis.

### Heterozygous Deletion of A20 Results in Myeloid-Biased Hematopoiesis

**Figure 2:**
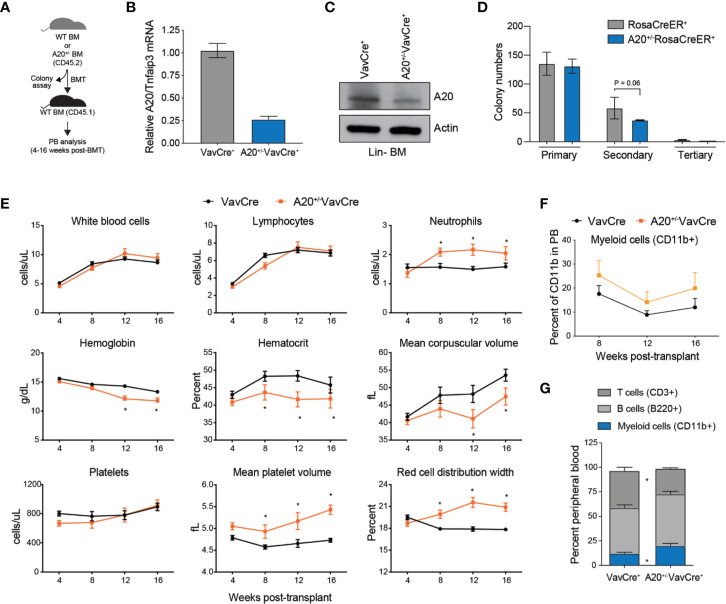
Heterozygous deletion of A20 results in myeloid-biased hematopoiesis. **(A)** Outline of experiments utilizing WT (VavCre^+^ or RosaCreER^+^) and A20^+/-^ (A20^+/-^VavCre^+^ or A20^+/-^RosaCreER^+^) mice for colony progenitor function and PB analysis. **(B)** A20 mRNA expression by qRT-PCR in lineage negative BM (Lin- BM) cells from VavCre+ and A20+/-VavCre+ mice (n = 3 mice per group). **(C)** A20 protein expression by immunoblotting in Lin- BM cells from VavCre+ and A20+/-VavCre+ mice (pooled from two mice). (**D**) Colony formation in methylcellulose of BM LSK cells isolated from WT RosaCreER^+^ and A20^+/-^RosaCreER^+^ mice (treated with tamoxifen). One-tailed T test from three mice per group. **(E)** Complete blood counts for WT (VavCre^+^; n = 3) and heterozygous A20-deficient (A20^+/-^VavCre^+^; n = 6) mice at the indicated time points post BM transplantation. *P < 0.05. **(F)** Flow cytometric analysis of donor-derived (CD45.2^+^) myeloid cells (CD11b^+^) in the PB from recipient mice reconstituted with BM from VavCre^+^ (n = 7) or A20^-/-^VavCre^+^ BM cells (n = 7) (CD45.2^+^) at the indicated time points post-transplantation. **(G)** Flow cytometric analysis of donor-derived (CD45.2^+^) myeloid cells (CD11b^+^) and lymphoid cells (CD3^+^ and B220^+^) in the PB from recipient mice reconstituted with BM from VavCre^+^ or A20^-/-^VavCre^+^ BM cells (CD45.2^+^) at 16 weeks post-transplantation.

Since A20 expression is reduced approximately by 40%–50% in aged HSC, we decided to examine the hematopoietic phenotype in mice reconstituted with BM cells with heterozygous deletion of A20 (**Figure 2A**). To acquire A20^+/-^ BM cells, we crossed *A20-*floxed mice with either VavCre^+^ or RosaCreER^+^ transgenic mice. Partial reduction by ~75% in A20 expression was confirmed in Lin- BM cells isolated from A20^+/-^VavCre^+^ mice or A20^+/-^RosaCreER^+^ mice (following treatment with tamoxifen) by qRT-PCR to detect *A20* mRNA, and by immunoblotting to detect loss of A20 protein expression (**Figures 2B, C**). In contrast to A20^-/-^VavCre^+^ mice (**Supplemental Figure 2A**), mice with deletion of one A20 allele (A20^+/-^VavCre^+^) were born at normal mendelian ratios, exhibited no signs of overt disease, and were indistinguishable from wildtype littermate controls (**Supplemental Figure 3**). Moreover, progenitor colony formation in methylcellulose was similar between A20^+/-^ (A20^+/f^RosaCreER^+^ upon treatment with tamoxifen) and WT (RosaCreER^+^) BM cells after the primary plating, but a modest reduction in colony re-plating of A20^+/-^ LSK cells as compared to WT LSK cells (P = 0.06)(**Figure 2D**). To determine the consequences of heterozygous A20 deletion on hematopoiesis, BM cells isolated from WT (VavCre^+^) and A20^+/-^VavCre^+^ were transplanted into lethally irradiated syngeneic recipient mice (**Figure 2A**). Unlike recipient mice transplanted with A20^-/-^VavCre^+^ BM cells (**Supplemental Figure 2E**), recipient mice reconstituted with A20^+/-^VavCre^+^ BM cells survived beyond 14 days, indicating that partial expression of A20 is sufficient for short-term hematopoiesis in lethally irradiated mice (data not shown). Moreover, the overall long-term survival of mice reconstituted with A20^+/-^VavCre^+^ BM cells was similar to WT (VavCre^+^) mice (data not shown). Although partial loss of A20 expression in hematopoietic cells is compatible with overcoming the hematopoietic deficient following myeloablation, we observed that mice reconstituted with A20^+/-^VavCre^+^ BM cells acquired changes in blood formation. Serial peripheral blood counts revealed that mice reconstituted with A20^+/-^VavCre^+^ BM cells exhibited normal total white blood cells (WBC), lymphocytes, and platelets (**Figure 2E**). However, mice reconstituted with A20^+/-^VavCre^+^ BM cells had increased neutrophils compared to control transplanted mice (2,040 vs. 1,580 cells/µl) (**Figure 2E**). Furthermore, hemoglobin (11.76 vs. 13.31 g/dl), mean corpuscular volume (47.47 vs. 53.58 fl), and hematocrit (41.83 vs. 45.77 percent) were trending lower in mice engrafted with A20^+/-^VavCre^+^ BM cells after 16 weeks (**Figure 2E**). Interestingly, red blood cells were roughly equivalent over the 16 weeks. Although mice transplanted with A20^+/-^VavCre^+^ and WT BM cells had equal numbers of platelets, the platelets in mice transplanted with A20^+/-^VavCre^+^ BM cells were larger (5.43 vs. 4.73 fl) (**Figure 2E**). Consistent with increased neutrophil counts, the proportion of CD11b^+^ myeloid cells (P = 0.016) in the peripheral blood was increased while the proportion of CD3^+^ T cells (P = 0.008) and B220^+^ B cells (P = 0.17) were reduced in the PB of A20^+/-^VavCre^+^ mice as compared to control VavCre^+^ mice (**Figures 2F, G**). These findings indicate that partial reduction of A20 results in myeloid-biased hematopoiesis, a phenotype also observed in aged mice.

### Heterozygous Deletion of A20 Affects Hematopoietic Stem and Progenitor Cell Frequency

**Figure 3:**
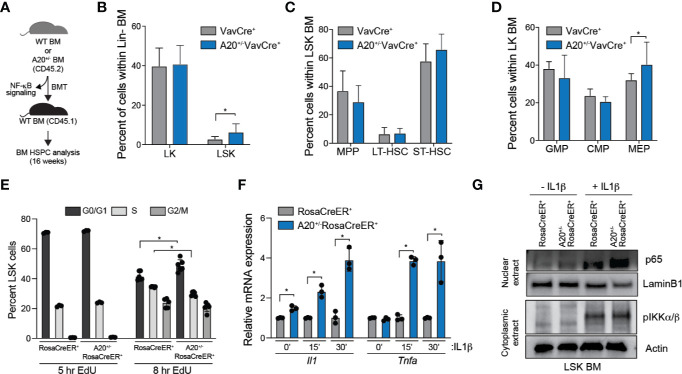
Heterozygous deletion of A20 affects hematopoietic stem and progenitor cell frequency. **(A)** Outline of experiments utilizing WT (VavCre^+^ or RosaCreER^+^) and A20^+/-^ (A20^+/-^VavCre^+^ or A20^+/-^RosaCreER^+^) mice for NF-κB signaling and HSPC analysis. **(B–D)** Summary of BM HSPC proportions at 16 weeks after transplantation using VavCre^+^ (n = 6) or A20^+/-^VavCre^+^ BM cells (n = 8). **(E)** Flow cytometric analysis of RosaCreER^+^ and A20^+/-^RosaCreER^+^ LSK BM treated with 10 μM EdU for 5 h (LSK pooled from three mice per group) or 8 h (LSK from three individual mice per group) followed by staining with anti-EdU and DAPI. **(F)** *Il1* and *Tnfa* mRNA expression by qRT-PCR in RosaCreER^+^ and A20^+/-^RosaCreER^+^ LSK with 10 μM IL-1β stimulation for the indicated lengths of time (n = 3 mice per group). **(G)** Expression of nuclear p65 and cytoplasmic pIKKα/β by immunoblot in RosaCreER^+^ and A20^+/-^RosaCreER^+^ LSK BM cells with and without 15 min 10 μM IL-1β treatment. *P < 0.05. Data are represented as mean ± SEM.

During aging, there is an observed increase of HSC frequency yet a functional decline in regeneration potential and fitness of HSC. Complete deletion of A20 results in expansion and then a rapid attrition of HSCs and HSPCs under stressed conditions, such as following reconstitution of lethally irradiated mice. To examine the consequence of partial loss of A20 on HSPC frequency and regenerative potential, BM cells from A20^+/-^VavCre^+^ and WT VavCre^+^ mice were transplanted into lethally irradiated recipient mice and BM HSPC populations were examined after 16 weeks (**Figure 3A**). The A20^+/-^VavCre^+^ transplanted mice had a significant increase in proportion of CD45.2^+^ LSK cells compared to wildtype (**Figure 3B**). Within the LSK compartment, A20^+/-^VavCre^+^ transplanted mice had decreased multipotent progenitor (MPP) cells and increased short-term hematopoietic cells (ST-HSC) (**Figure 3C**, **Supplemental Figure 4**). Further analysis of the Lin^-^c-Kit^+^ (LK) cells revealed no significant differences in the progenitor populations, however, mice transplanted with A20^+/-^VavCre^+^ BM cells had a moderate decrease in granulocyte monocyte progenitor (GMP) and common myeloid progenitor (CMP) populations accompanied by an increase in megakaryocyte erythroid progenitor (MEP) cells (**Figure 3D**). Differences in apoptosis, as measured by Annexin V staining (**Supplemental Figure 5A**), were not observed between WT (RosaCreER^+^) and A20^+/-^ LSK (A20^+/-^RosaCreER^+^). However, we observed a modest yet significant increase of A20^+/-^ LSK in the G0/G1 phase and a corresponding decrease in the S phase of the cell cycle as compared to WT LSK as measured by 5-ethynyl-2’-deoxyuridine (EdU) uptake after 8 h (**Figure 3E**). The reduction in EdU uptake A20^+/-^ LSK was confirmed by examining cell proliferation *in vitro* over 7 days (**Supplemental Figure 5B**). Since A20 can restrict inflammatory signaling *via* NF-κB, we examined cytokine gene expression and NF-κB activation in response to interleukin 1β (IL-1β) stimulation *in vitro*. A20^+/-^ (A20^+/-^RosaCreER^+^) LSK exhibited a significant increase in cytokine gene expression (*Il1a* and *Tnfa*) upon IL-1β stimulation compared to WT (RosaCreER^+^) LSK (**Figure 3F**). Since A20 negatively regulates NF-κB and a hallmark of NF-κB activation is its translocation to the nucleus, we performed subcellular fractionation of WT (RosaCreER^+^) and A20^+/-^ (A20^+/-^RosaCreER^+^) LSK with and without IL-1β stimulation. While the abundance of nuclear p65, an NF-κB subunit, was equivalent in the two groups under unstimulated conditions (-IL1β), nuclear p65 abundance was increased in A20^+/-^ upon cytokine stimulation (+IL1 β) (**Figure 3G**). Given that the *in vivo* environment includes exposure to inflammatory stimuli, the diminished ability to resolve NF-κB signaling may explain the impaired function of aged HSCs with reduced A20 expression.

**Figure 4:**
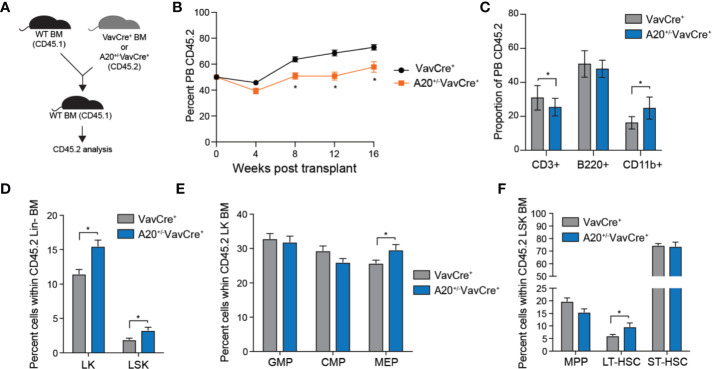
Heterozygous deletion of A20 results in a competitive advantage of hematopoietic stem and progenitor cells. **(A)** Outline of competitive BM transplantations using VavCre^+^ or A20^+/-^VavCre^+^ mice. **(B)** Summary of donor-derived PB proportions at the indicated time points after competitive transplantation using VavCre^+^ or A20^+/-^VavCre^+^ BM cells (n = 10 per group). **(C)** Proportion of donor-derived VavCre^+^ or A20^+/-^VavCre^+^ myeloid (CD11b^+^) and lymphoid (CD3^+^ and B220^+^) proportions at 16 weeks following competitive transplantation (n = 9 per group). **(D-F)** Summary of donor-derived BM HSPC proportions at 16 weeks after competitive transplantation using VavCre^+^ or A20^+/-^VavCre^+^ BM cells (n = 9 per group). *P < 0.05. Data are represented as mean ± SEM.

To further investigate the consequences of partial A20 deficiency on HSC function, we performed competitive BM transplantation assays. Equal numbers of BM cells from A20^+/-^VavCre^+^ or VavCre^+^ mice (CD45.2^+^) were mixed with WT competitor cells (CD45.1^+^), transplanted into lethally-irradiated congenic recipient mice, and then donor-derived (CD45.2^+^) hematopoietic reconstitution was determined by FACS (**Figure 4A**). The hematopoietic contribution of A20^+/-^VavCre^+^ BM cells to PB chimerism was moderately reduced (from ~70% to 50%) compared with WT (VavCre^+^) donor BM cells (**Figure 4B**). The changes in PB chimerism of A20^+/-^VavCre^+^ BM cells at 16 weeks post transplantation was a result of reduced lymphoid (CD3^+^) cells albeit an increase in myeloid cells (CD11b+) as compared to mice reconstituted with WT (VavCre^+^) BM cells (**Figure 4C**). BM chimerism of donor-derived LSK (P = 0.010), LK (P = 0.002), MEP (P = 0.038), and LT-HSC (LSKCD150^+^CD48^-^; P = 0.035) was significantly increased in mice engrafted with A20^+/-^VavCre^+^ BM cells as compared to control donor BM cells 4 months post transplantations (**Figures 4D–F**). Collectively, these observations suggest that partial reduction of A20 contributes to phenotypic and functional changes in hematopoietic cells, including increased HSPC frequency, decline in regenerative potential, and myeloid-biased hematopoiesis as observed during aging.

## Discussion

We report that aged HSCs express lower levels of A20, a critical regulator of inflammation, and that partial reduction in A20 expression in young HSCs results in characteristic features of hematopoietic aging. Specifically, heterozygous deletion of A20 in hematopoietic cells resulted in expansion of the HSC pool, reduced HSPC fitness, and myeloid-biased hematopoiesis. Interestingly, a recent analysis identified frequent loss of one copy of TNFAIP3/A20 in aged healthy individuals of Japanese descent (BioBank Japan) with clonal hematopoiesis (34). This observation is consistent with our findings that revealed heterozygous deletion of A20 results in a competitive advantage and expansion of HSPCs, albeit these A20-deleted HSPCs exhibit myeloid-biased hematopoietic output. Since HSC experience a functional decline in response to chronic inflammation or aging, our findings point to the potential role of A20 as a common link between inflammatory states and aging. Under normal cellular conditions, A20 is expressed at low levels, however, stimulation of PAMPs and pro-inflammatory cytokines acutely induce the expression of A20 in an NF-κB-dependent manner (19, 20). In response to several immune pathways including Tumor Necrosis Factor Receptor, Interleukin-1 Receptor, and Toll-Like Receptor signaling, A20 negatively regulates excessive NF-κB signaling by modifying ubiquitin chains on key intermediates RIP1 and TRAF6 (16–18). The most striking data to demonstrate the involvement of A20 in the innate immune system is that A20-deficient mice prematurely die of systemic inflammation (24). Moreover, putative causal single nucleotide polymorphisms (SNPs) in the vicinity of A20 affect its expression and are associated with immune disorders (35–40). Given that defects in A20 are linked to the pathogenesis of autoimmune disorders and hematologic malignancies (13, 21), we posited that partially reduced levels of A20 may be playing an important role in the phenotypes associated with aged HSCs. Our findings revealed that epigenetic suppression of A20 in HSPCs leads to hematopoietic changes associated with an aging phenotype. Moreover, the negative feedback loop of suppressing NF-κB and associated inflammatory signaling by A20 is not functional in aged HSC.

A20 is a dual-ubiquitin modifying enzyme. The dual ubiquitin-editing functions of A20 are responsible for the restriction of multiple intracellular signaling cascades, including those of NF-κB and RIP. One of its defined functions is to remove lysine (K) 63-linked ubiquitin chains, *via* its deubiquitinase domain, from TRAF6, a signaling mediator upstream of NF-κB. In the absence of A20, TRAF6 undergoes K63-linked ubiquitination, which results in NF-κB activation (17, 41). Interestingly, TRAF6 is overexpressed in HSC from myelodysplastic syndrome (MDS) patients as compared to healthy individuals (42, 43), and chronic innate immune signaling *via* TRAF6 has been implicated in hematopoietic defects associated with MDS (31, 43–47). Utilizing a transgenic mouse model, TRAF6 overexpression in hematopoietic cells results in a competitive disadvantage, increased HSPC frequency, and myeloid-biased differentiation (42). Intriguingly, several of the hematopoietic phenotypes observed in TRAF6-overexpresing HSPCs overlap with those observed in A20 heterozygous deficient HSPCs. Although NF-κB and immune signaling is associated with normal TRAF6 function, gene expression and molecular analyses indicated that immune and inflammatory responses were not upregulated in TRAF6-overexpressing HSC (42). However, we observed that activation of the small RhoGTPase Cdc42 was largely responsible for the hematopoietic phenotype exhibited by mice that express TRAF6 in HSPCs (42). Elevated Cdc42 activation has been causally linked to ineffective HSC function upon aging (48). Based on these findings, it is likely that TRAF6 overexpression alone is not sufficient to induce NF-κB, but rather environmental stressors are needed to augment TRAF6-induced NF-κB activation in HSC. Indeed, baseline activation of canonical NF-κB signaling, as indicated by nuclear translocation of p65/RelA, was comparable in WT and A20^+/-^ HSPC; however, NF-κB activation and inflammatory gene expression were significantly elevated in A20^+/-^ HSPCs following IL-1β stimulation (**Figures 3F–G**). In a recent study by Muto et al, it was shown that HSPCs poised for TRAF6 signaling have a competitive advantage over WT HSPCs during low-grade chronic inflammation resulting from increased non-canonical NF-κB activation (49). However, in these series of studies, TRAF6-induced A20 expression resulted in diminished canonical NF-κB activation and contributed to increased non-canonical NF-κB activation. The precise mechanism by which A20 loss may contribute to an aged HSC phenotype, particularly in an aged and inflamed BM microenvironment, is not entirely clear. Although, one potential reason for the hematopoietic defects observed in heterozygous A20 deleted cells is due to unrestricted TRAF6 signaling, Cdc42 activation, and/or NF-κB activation. Nevertheless, these findings suggest that the negative feedback loop of suppressing canonical NF-κB and associated inflammatory signaling by A20 is not intact in aged HSC. Future studies are needed to understand the transcriptional mechanisms by which A20 expression is regulated in young versus aged HSC, determine the precise mechanism resulting from reduced A20 expression, such as identification of the relevant ubiquitinated substrates and downstream pathways, and explore the potential of restoring A20 expression to rejuvenate aged HSC.

## Data Availability Statement

Data can be found here: GSE104404. Other raw data supporting the conclusions of this article will be made available by the authors, without undue reservation, to any qualified researcher.

## Ethics Statement

Animals were bred and housed in the Association for Assessment and Accreditation of Laboratory Animal Care-accredited animal facility of Cincinnati Children’s Hospital Medical Center. All experiments conform to the regulatory standards of the Institutional Animal Care and Use Committee (IACUC) and adhere to IACUC-approved protocols. The study was approved by the IACUC ethics committee at Cincinnati Children’s Hospital Medical Center.

## Author Contributions

Study conception and design: MS, AC-C, and DS. Acquisition of data: AC-C, MS, GR, and EA. Analysis and interpretation of data: MS, GR, EA, AC-C, DS, and MF. Writing of the manuscript: MS, AC-C, DS, and MF. Review of the manuscript: MS, AC-C, EA, GR, MF, DS, and AM. Administrative, technical, or material support: AM. All authors contributed to the article and approved the submitted version.

## Funding

This work was supported in parts by the National Institutes of Health (R35HL135787, R01DK102759, R01DK113639 to DS and R01HL126947 to MF), Leukemia and Lymphomas Society (DS), and Cincinnati Children’s Hospital Research Foundation (DS). MS was supported by a National Institute of Health Research Training and Career Development Grant (F31HL132420). AC-C is supported by a National Institutes of Health Research Training and Career Development Grant (F32CA232402) and EA is supported by a T32CA217835 training award from the National Institutes of Health. DS and MF are Leukemia and Lymphoma Society Scholars.

## Conflict of Interest

DS is a consultant for Kurome Therapeutics.

The remaining authors declare that the research was conducted in the absence of any commercial or financial relationships that could be construed as a potential conflict of interest.
