# Supplementary material for: TNFAIP3 Plays a Role in Aging of the Hematopoietic System
Source: Front Immunol. 2020 Nov 3;11:536442. doi: 10.3389/fimmu.2020.536442 (PMC7670064; doi:10.3389/fimmu.2020.536442)
Supplement: Supplementary file 1 [file DataSheet_1.pdf]

Supplemental Figure 1

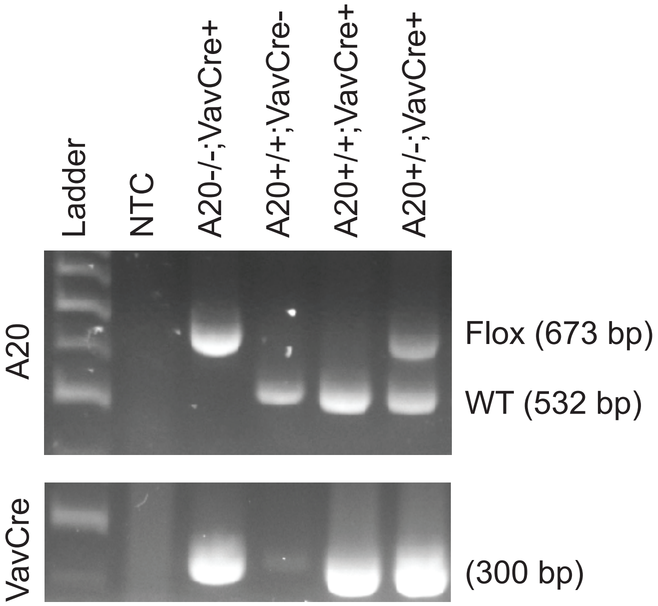

Supplemental Figure 1. Outcome of genetic crossing of A20 floxed and VavCre mice. Genotyping of A20 and VavCre in mouse tail DNA. NTC; no template control.

Supplemental Figure 2

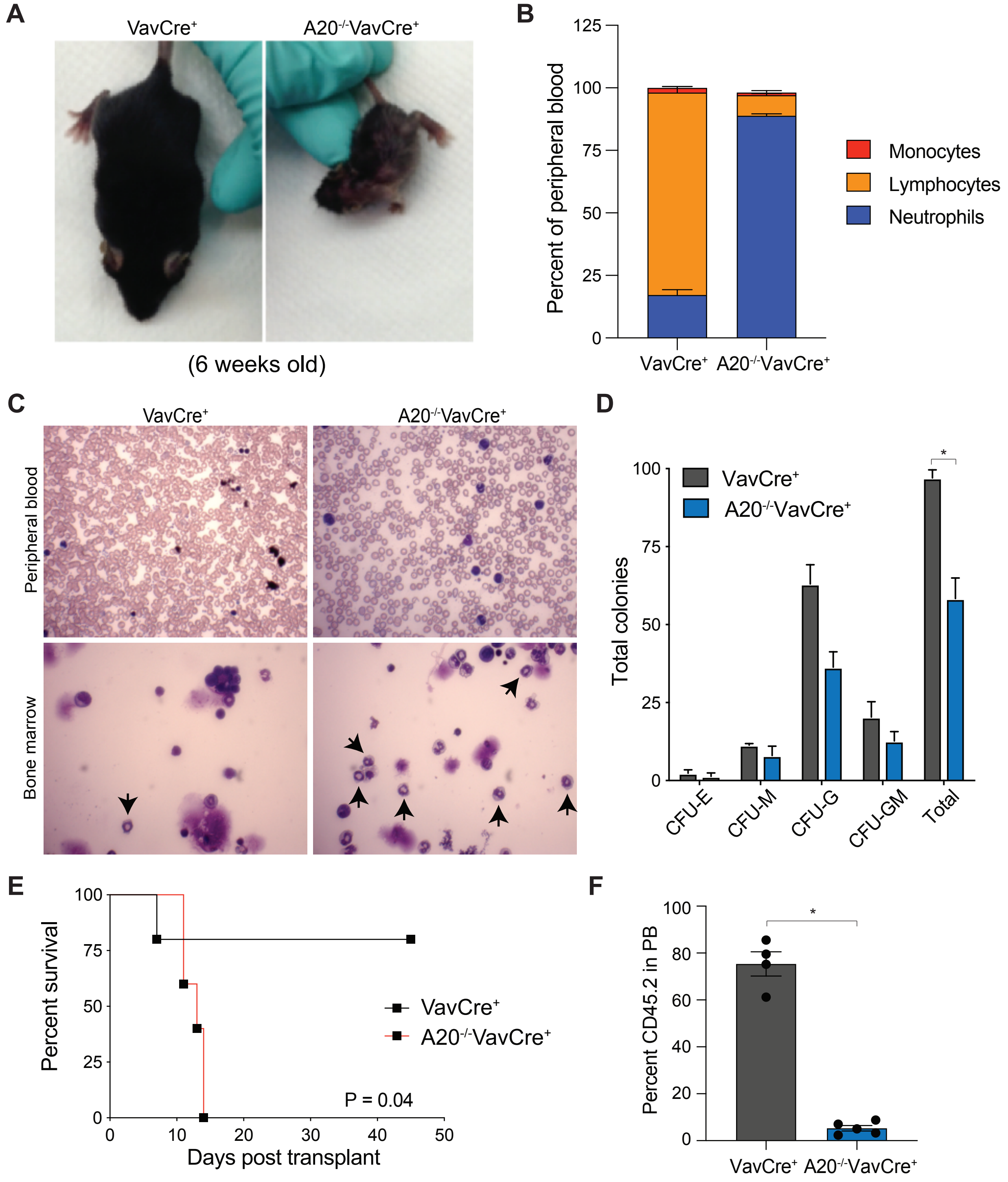

Supplemental Figure 2. Homozygous deletion of A20 impairs normal HSC function and hematopoiesis. (**A**) Representative image of 4-week old WT (VavCre+) and homozygous A20-deficient (A20-/-VavCre+) mice. (**B**) Complete blood counts for WT (VavCre+; n = 5) and A20-deficient (A20-/-VavCre+; n = 3) mice at ~8 weeks of age. (**C**) Representative images of Wright-Giemsa stained peripheral blood (PB) smears and BM cytospins from VavCre+ and A20-/-VavCre+ mice. (**D**) Colony formation in methylcellulose of BM mononuclear cells isolated from VavCre+ and A20-/-VavCre+ mice. (**E**) Overall survival of lethally irradiated mice transplanted with BM cells from VavCre+ (n = 5) and A20-/-VavCre+ (n = 5) mice. (**F**) Flow cytometric analysis of donor-derived (CD45.2+) and competitor-derived (CD45.1+) PB from recipient mice after competitive transplantation using VavCre+ or A20-/-VavCre+ BM cells (CD45.2). Shown are the percent of donor-derived VavCre+ or A20-/-VavCre+ cells in the PB ~12 weeks post transplantation.

Supplemental Figure 3

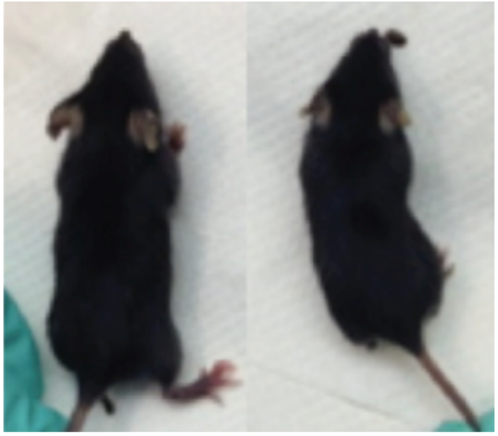

Supplemental Figure 3. Mice with deletion of one A20 allele were indistinguishable from wildtype littermate controls. Representative image of 4-week old WT (VavCre+) and heterozygous A20-deficient (A20+/-VavCre+) mice.

Supplemental Figure 4

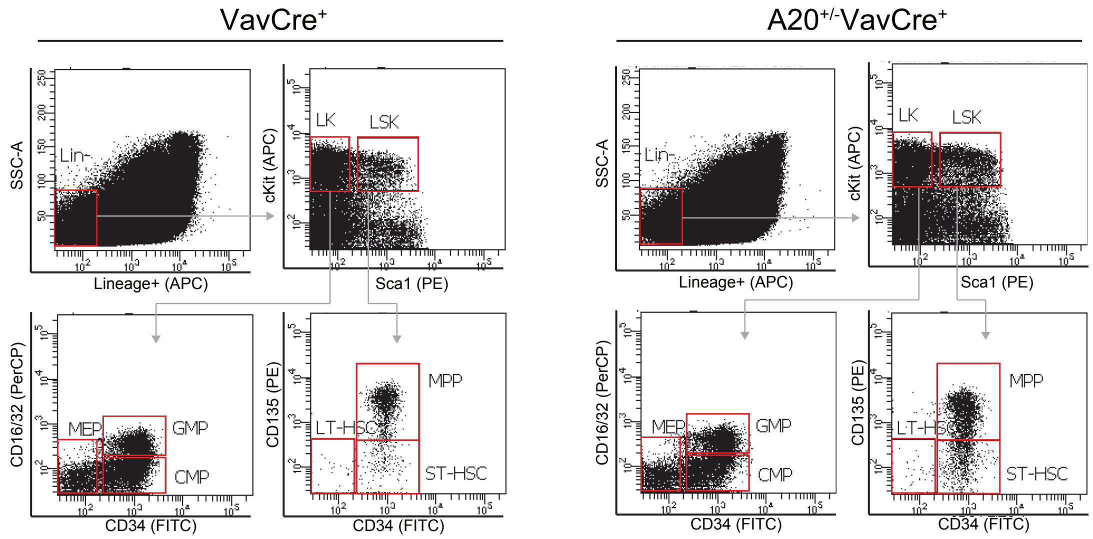

Supplemental Figure 4. Gating strategy of HSPCs isolated from the BM of recipient mice. Representative image of 16-week old WT (VavCre+) or heterozygous A20-deficient mice (A20+/-VavCre+).

Supplemental Figure 5

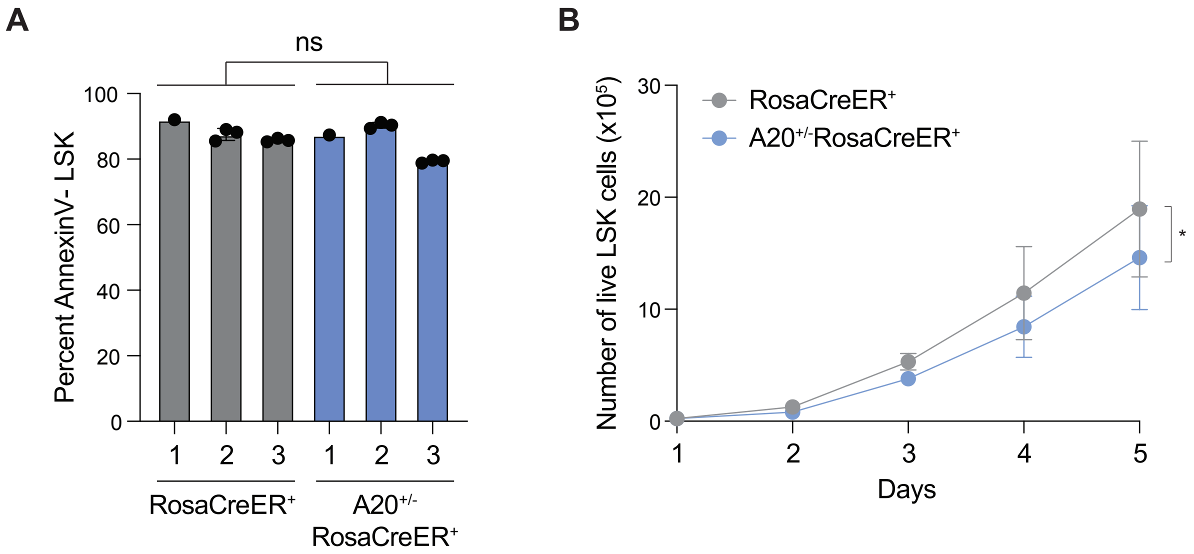

Supplemental Figure 5. Apoptosis and cell proliferation analysis of A20 heterozygous-deleted HSPCs. (**A**) AnnexinV staining of LSK cells isolated from the BM of the indicated mice (3 independent mice per group examined in triplicate: #1-3) after treatment with Tamoxifen. (**B**) Viable cell counts (trypan blue exclusion) of LSK cells isolated from the BM of the indicated mice (n = 3 mice per group) after treatment with Tamoxifen. *, $P < 0.05$.
